# Using the ages and stages questionnaire in the general population as a measure for identifying children not at risk of a neurodevelopmental disorder

**DOI:** 10.1186/s12887-018-1105-z

**Published:** 2018-04-03

**Authors:** Ramesh Lamsal, Daniel J. Dutton, Jennifer D. Zwicker

**Affiliations:** 1 School of Public Policy, University of Calgary, 906 8th Ave SW, Calgary, Canada; 2 Faculty of Kinesiology, University of Calgary, 2500 University Drive NW, Calgary, AB T2N 1N4 Canada

**Keywords:** Ages and stages questionnaire (ASQ), Neurodevelopmental disabilities, Early identification, Early intervention, Screening

## Abstract

**Background:**

Early detection of neurodevelopmental disorders (NDDs) enables access to early interventions for children. We assess the Ages and Stages Questionnaire (ASQ)’s ability to identify children with a NDD in population data.

**Method:**

Children 4 to 5 years old in the National Longitudinal Survey of Children and Youth (NLSCY) from cycles 5 to 8 were included. The sensitivity, specificity, positive and negative predictive values were calculated for the ASQ at 24, 27, 30, 33, 36 and 42 months. Fixed effects regression analyses assessed longitudinal associations between domain scores and child age.

**Results:**

Specificity for the ASQ was high with 1SD or 2SD cutoffs, indicating good accuracy in detecting children who will not develop a NDD, however the sensitivity varied over time points and cut-offs. Sensitivity for the 1 SD cutoff at 24 months was above the recommended value of 70% for screening. Differences in ASQ domains scores between children with and without NDD increases with age.

**Conclusions:**

The high specificity and negative predictive values of the ASQ support its use in identifying children who are not at the risk of developing a NDD. The capacity of the ASQ to identify children with a NDD in the general population is limited except for the ASQ-24 months with 1SD and can be used to identify children at risk of NDD.

**Electronic supplementary material:**

The online version of this article (10.1186/s12887-018-1105-z) contains supplementary material, which is available to authorized users.

## Background

Early childhood is a uniquely sensitive period for developing cognitive ability, language, social and motor development. Both developmental delay and neurodevelopmental disorders (NDDs) manifest early in life. While, developmental delays signifies a failure to meet developmental milestones for children under the age of five [1], NDDs are characterized as life-long disabilities associated with poorer long-term health outcomes [2], quality of life implications for children and their families [3–5] and higher healthcare and societal costs [6]. The prevalence of NDDs in children is estimated to range from 5 to 9%, depending on the definition and data set used [7–9].

Early detection and intervention for children with developmental delays or disorders, is recognised as an essential part of good health care to optimise outcomes for children and families [10, 11]. The successful identification of children with a NDD requires two stages: screening for risk of developmental delays using a valid and reliable screening tool and a comprehensive diagnostic evaluation of children whose development differs from the same age-norms [12]. The Canadian Paediatric Society supports developmental screening of all children at 18 months using a validated screening tool [13], and the American Academy of Pediatrics recommends developmental screening at 9, 18 and 24 or 30 months [12]. While a diagnosis by an experienced health care professional could occur by the age of two, many children do not receive a diagnosis until after the age of four [14–16]. In Canada, a study modelling time to diagnosis found that socioeconomic and sociodemographic factors had little impact [17]. This study suggests health system characteristics and differing wait times in regions may be key factors in time to diagnosis. This delay in diagnosis limits access to early intervention, as children with developmental delays may not receive early intervention services until their delays manifest more profoundly to affect functional abilities or when they receive a diagnosis [18]. There is evidence to suggest that protective factors such as social support and community engagement for children at risk could include low cost identification and intervention strategies in the community with parents to decrease the risk for child developmental and behavioural problems [19].

The use of parent-completed screening tools, such as the Ages and Stages Questionnaire (ASQ), has increased in recent years because of being a low-cost screening tool with a short completion time, easily administered by parents in the home setting. The ASQ has been shown to perform well in children with biological risk factors [20–22] or some environmental risk factors [23]. As a parent report tool, it provides an important communication opportunity for parents to discuss concerns they have about their child’s development in a structured manner with a healthcare professional [24].

The original ASQ has been proven to be reliable and valid with an overall sensitivity of 75% and specificity of 86% in detecting developmental delays, but it is unclear how many children go on to have a developmental disability [25]. Many of the validation studies to date are in high-risk children in clinical settings [26–28]. This study uses population data to look at the validity of the ASQ as a screening tool for identifying children at risk of a developmental delay who are later identified in the data as having a NDD. The objectives of our study were 1) to evaluate the ability of the ASQ questionnaire to identify children with and without a NDD using population-based survey data and 2) to analyse and compare ASQ domain scores for children with and without a NDD different time points.

## Methods

### Data source

This study used data from the National Longitudinal Survey of Children and Youth (NLSCY), a long-term study of the physical and social development of Canadian children from birth to early adulthood [29]. The survey conducted jointly by Statistics Canada and Human Resources Development Canada began in 1994 and was repeated biennially. Data collection stopped in cycle 8 (2009). The target population of the survey comprised the noninstitutionalized civilian population aged 0 to 11 months at the time of data collection. The person most knowledgeable (PMK), usually mothers, provided information on the selected child. More details about data collection can be found in the Statistics Canada user guide [29].

### Ages and stages questionnaire (ASQ)

ASQ is a standardised parent completed questionnaire to screen for developmental delays in children using 19 age-specific developmental questionnaires starting at 4 months and ending at 60 months of age [25]. The ASQ-2 was the version used in NLSCY data collection. There are five domains: Fine Motor, Gross Motor, Communication, Problem-Solving and Personal-Social; each domain contains six questions that can be answered with a yes (10 points), sometimes (5 points) or not yet (0 points), as well as nine open-ended questions. Scores obtained from each domain are compared with established cut-off points at one and two standard deviations that are used to identify children at risk of developmental delay. If the score on any domain falls below the 2SD cut-off, referral for further assessment is advised. If the score on any domain is within the one standard deviation (1SD) and 2SD cut-off point, it is advised to provide learning activities and monitor the child’s development.

### Identification of children with neurodevelopmental disorders

Children with NDD were identified using two indicators: (a) a parent reported checklist of chronic conditions diagnosed by a health professional (for children 4 to 5 years old) and (b) health utility index-3(HUI) scores when children were 4 to 5 years old. The checklist included five chronic conditions: Cerebral Palsy, Epilepsy, Mental Handicap, Learning Disability, and Attention Deficit Disorder (and Autism in cycle 8). The HUI is a generic preference-based instrument used to measure overall health status and health-related quality of life (HRQoL) of life of individuals [30]. It includes eight domains-vision, hearing, speech, ambulation, dexterity, emotion and pain. Cut-off values for four domains of the HUI established by Lach et al. [8] for identifying children with NDD were used: > 3 for the speech and mobility domains, > 2 for the dexterity domain, and > 4 for the cognition domain. If one of the indicators identified children, they were classified as children with NDD and were combined to obtain the sample.

Unfortunately, the gross motor portion of the ASQ was not included in cycle 5 and onwards, so we were limited to use only four domains- Fine Motor, Communication, Problem-Solving, and Personal-Social [29].

### Sample

The sample was restricted to children aged 4 to 5 years in cycles 5 to 8 because the HUI was only collected for children 4 to 5 years old in the survey. Two cohorts were created to address the research objectives: the short cohort for objective 1 and the long cohort for objective 2 (Additional file 1). The short cohort consisted of children who were 4 to 5 years old in cycles 5, 6, 7, or 8, and was also present in the corresponding previous cycle. Observations from different cycles were aggregated into a single sample. For the short cohort (unweighted *n* = 12,142), 725 children with a NDD were identified. This sample for the short cohort was used to estimate sensitivity, specificity, positive predictive value (PPV) and negative predictive value (NPV) of the ASQ. The long cohort consisted of 350 children with a NDD (5604 total observations) who were 4 to 5 years old in cycles 5, 6, 7, or 8, and was also present in the corresponding two previous cycles. This sample was used to compare the ASQ domain scores longitudinally using fixed effects regression modelling.

Weighted proportions of the sample size for the short cohort and the long cohort can be found in Table 1. Using both the HUI and the checklist of chronic conditions, 6.47% of children aged 4 to 5 years were identified as having NDD for the short cohort (HUI 5.65% and reported 1.88%). The percentage was slightly higher 7.17% for the long cohort (HUI 6.26% and reported 1.91%). The percentage of children reported to have a learning disability was higher, and epilepsy was lower compared to other NDDs in both cohorts.

**Table 1:** Weighted Proportion of Sample Size for the Short Cohort and the Long Cohort

	Short Cohort (unweighted *n* = 12,142)	Long Cohort (unweighted *n* = 5604)
Health Utilities Index (HUI)	5.65%	6.26%
Reported diagnosis	1.88%	1.91%
Epilepsy	0.19%	0.15%
Cerebral Palsy	0.22%	0.23%
Mental Handicap	0.27%	0.30%
Learning Disability	1.11%	1.17%
Attention Deficit Disorder	0.64%	0.66%
Autism^b^	0.50%	0.49%
Both	0.99%	0.96%
NDD^a^	6.47%	7.17%

^a^Some children are diagnosed with more than one NDD

^b^Based on cycle 8 sample only

### Data analyses

Descriptive statistics were generated for children with and without NDD at baseline. Two-sample t-tests (age and HUI) and two-sample tests of proportions (income, gender, race, and education) were conducted to examine group differences. The screening accuracy of ASQ was determined by comparing passed/failed status on domains for children with and without a NDD. The passed/failed status was defined by using the established age-specific cut-off scores for each domain i.e. less than 1SD/2SD below the mean in any domain was defined as a failure [25]. Next, sensitivity, specificity, PPV, and NPV were calculated for 1SD and 2SD of ASQ at 24, 27, 30, 33, 36 and 42 months.

Fixed effects regression analyses were conducted to examine longitudinal associations between domain scores and age of children accounting for the effects of other domains. The models were analysed separately for each domain (communication, personal social, fine motor and problem-solving) for children with and without NDD. For example, the fixed effect model for the fine motor domain was:

$$ {\mathrm{Finemotor}}_{it}={\upalpha}_i+{\upbeta}_1{\mathrm{Agemonths}}_{it+}{\upbeta}_2\ {\mathrm{Communication}}_{it+}{\upbeta}_3\ {\mathrm{Problemsolving}}_{it}\kern0.75em +{\upbeta}_4\ {\mathrm{Personalsocial}}_{it+}\ {\upvarepsilon}_{it,} $$

where α_*i*_ (*i =* 1…..n) is the individual-level fixed effect. Finemotor_*it*_ is the dependent variable where *i* = child, *t* = time, β is the coefficient for the respective variable and. Agemonths_*it,*_ Communication _*it,*_ Problemsolving_*it*_ and Personalsocial_*it*_ are independent variables, and ɛ_*it*_ is the error term.

All analyses were weighted (and bootstrapped for fixed effects models) to account for the complex survey design and to ensure that the sample size was representative of the Canadian population. Statistics Canada provides the survey weights and 1000 bootstrap sample weights for each cycle [29]. Statistical software STATA 14 was used to conduct the analysis.

## Results

### Screening accuracy of ASQ in identifying children at risk of NDD

Weighted descriptive statistics of the study population in the short cohort are presented in Table 2. The mean age for children in the sample with and without a NDD at baseline was 34.91 months and 34.50 months, respectively. Children with a NDD in the short cohort were more likely to be male, non-white, from a less educated family with lower household income, compared to children without a NDD. Boys (65.75%) were twice as likely to have a NDD compared to girls (34.25%). Approximately 25.12% of children with a NDD were from low-income households compared to 15.37% of children without a NDD. This sociodemographic information is in line with other studies finding children with NDD are more frequently male or from lower income families [9, 31–33].

**Table 2:** Weighted Demographic Characteristics for the Short Cohort and the Long Cohort

	Short Cohort		Long Cohort	
	NDD (unweighted *n* = 725)	Without NDD (unweighted *n* = 11,417)	NDD (unweighted *n* = 350)	Without NDD (unweighted *n* = 5254)
Age (months), mean (SD)	34.91(6.18)	34.50(6.11)	15.27(5.94)	14.80(5.96)
Gender^b^				
% Male	65.75	49.95	64.44	49.53
% Female	34.25	50.05	35.56	50.47
Education PMK^a^				
% Less than secondary	13.91	9.17	9.37	8.58
%Secondary school graduation	21.33	17.77	15.66	12.69
%Some post-secondary	13.68	12.29	14.79	13.57
%College or university or others	51.09	60.77	60.18	65.16
Race^a^				
%White	77.60	79.12	72.66	79.05
%Non-White	22.40	20.88	27.34	20.95
Income^a^				
% Low-income household	25.12	15.37	26.02	13.59
% Above low-income household	74.88	84.63	73.98	86.41
Relationships of PMK to the child^a^				
%Biological Mother/Father	96.55	98.63	96.19	98.76
%Others	3.45	1.37	3.18	1.24
Health Utilities Scores (HUI)				
mean(SD)^a^	0.68(0.31)	0.96(0.08)	0.67(0.34)	0.96(0.08)

Notes:

• Mean HUI scores for children who had a reported diagnosis of Autism, Cerebral Palsy, Mental Handicap, Learning Disability, Epilepsy and Attention Deficit Disorder

• The household income was based on the parent’s reported estimates for their household income. The household income was compared with the low-income cut-off (LICO) established by Statistics Canada and was considered to be low income when total household income is below the LICO cut-off for family size and community

^a^The difference between two groups was significant at 5% level

Health related quality of life (HRQoL) was significantly different for children with and without a NDD. PMK rated mean HUI scores for children with a reported NDD diagnosis to be 0.68 compared to 0.96 for children without a reported diagnosis. HUI scores can range from 0 to 1, ‘1’ as in perfect health and ‘0’ as in dead. The mean HUI scores for children diagnosed with epilepsy, attention deficit disorder, autism, learning disability, mental handicap and cerebral palsy were 0.75, 0.73, 0.72, 0.70, 0.32 and 0.31, respectively.

**Fig. 1:**
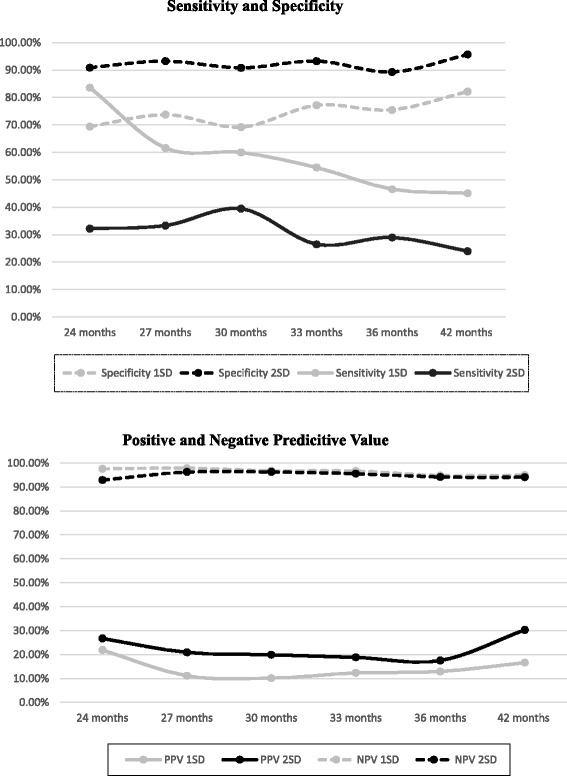
Sensitivity, Specificity, Positive Predictive and Negative Predictive values of ASQ for 1SD and 2SD at 24, 27, 30, 33, 36 and 42 months

The sensitivity, specificity, PPV and NPV of the ASQ at 24, 27, 30,33, 36 and 47 months can be seen in Fig. 1. When 1SD is used as the cut-off point, sensitivity at 24 months was 83.60% and dropped to 45.10% at 42 months. The specificity at 24 months was 69.40% and increased to 82.20% at 42 months. PPV for 1SD started at 21.90% for 24 months and dropped to 16.60% at 42 months. NPV for 1SD was 97.60% for 24 months and fell slightly to 95.00% at 42 months. Using a 2SD cut-off point the sensitivity at 24 months was 32.20% dropping to 24.00% at 42 months. The specificity for 2SD was 90.90% at 24 months increasing to 95.70% at 42 months. The PPV value for 2SD was 26.70% at 24 months and 30.30% at 42 months. NPV for 2SD was 92.90% at 24 months and increased to 94.10% for 42 months.

### ASQ domain scores over time for children with NDD and without NDD

The mean age for children at baseline who were later identified with and without a NDD in the long cohort was 15.27 months and 14.80 months, respectively (descriptive statistics in Table 2). Similar to the short cohort, children with NDD in the long cohort were more likely to be male, non-white and from a low-income household and less educated family compared to the children without NDD. The PMK rated mean HUI scores were 0.67 for children with a reported diagnosis with NDD and 0.96 for children without a reported diagnosis. The mean HUI scores for children diagnosed with epilepsy, attention deficit disorder, autism, learning disability, mental handicap and cerebral palsy were 0.71, 0.72, 0.75, 0.72, 0.22 and 0.26 respectively.

**Fig. 2:**
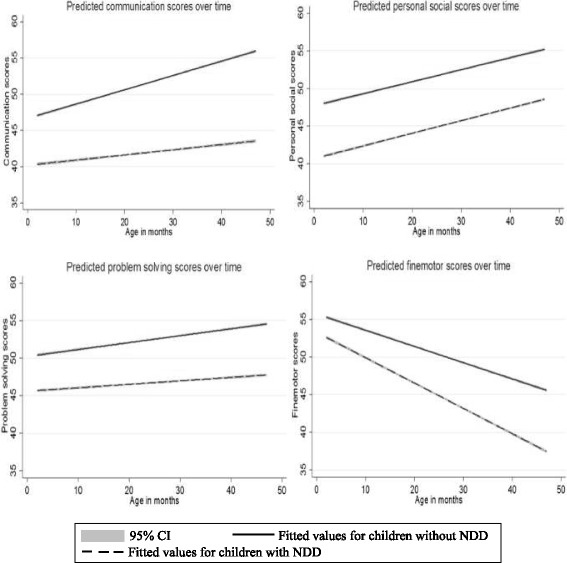
Predicted domains scores for children with NDD and without NDD over time from fixed effects regression. The models were analysed separately for each domain (communication, personal social, fine motor and problem-solving) for children with and without NDD accounting for the effects of other domains

We present predicted mean scores for each ASQ domain over time in Fig. 2. The communication, personal social and problem-solving domains increased as the age of children increased. The predicted mean scores for fine motor had a negative relationship with the age of children, which is consistent with the relationship between established cut-off values for this domain across ages [34]. Results from these fixed effect models demonstrated a strong correlation between domain scores and age of the children. For children without a NDD, the predicted mean scores for all four domains at baseline were higher compared to children with a NDD and similar trends can be observed over the time. For instance, the mean predicted communication scores for children with NDD at baseline was 40.65 and 46.89 for children without NDD. Mean fine motor scores were 52.65 and 55.43 for children with and without NDD, respectively.

## Discussion

The prevalence of children with NDD in our sample was 6% across our two cohorts. This finding supports earlier work using the NLSCY which estimated that 6% children between ages of 4 to 11 years have NDD [8]. While it is not possible to make a direct comparison with other published prevalence estimates owing to differences in data sources, age of children, and operationalization of the NDD definition, this percentage is within the range of other Canadian and international estimates [7–9, 31, 35, 36]. For example, a recent study using administrative data suggests 8.3% of 6–10-year-old children in British Columbia, Canada were identified as having a NDD [9].

The results from our study indicate that the ASQ specificity was high with 1SD or 2SD cut-offs, showing good accuracy in detecting children who will not develop a NDD. However, the sensitivity varied over time points and cut-offs. The sensitivity for 1 SD at 24 months (83.60%) was the only time point where the sensitivity was high enough to suggest it is an effective screening tool in the general population [12]. The NPV was high (92.90–97.60%) at all time points and for all cut-offs while the PPV was low (10.10–30.30%), reflecting the low prevalence of NDD in our sample. Thus, in general, ASQ was an accurate measure to identify children not at risk of NDD. The ASQ was not an effective screening tool for identifying children at risk of NDD, except when a 1SD cutoff was used at 24 months. When 1SD was used as a cut-off score, the ASQ had better test characteristics as a screening tool compared to 2SD.

Our study confirms findings from several other studies that reported the ability of the ASQ to exclude children without a NDD despite varying populations, settings and definitions used to identify children with a NDD [27, 37, 38]. Skellern et al. found that in general, the ASQ has a low sensitivity and high negative predictive value in detecting children with neurosensory disabilities [37]. They conducted their study in a clinical setting whereas our study was in a population-based sample and used a 2SD cutoff only. In contrast, using the same definition for classification of neurosensory disability for children in developing countries, Yu et al. found an acceptable adjusted sensitivity (87.4%) and specificity (82.3%) in detecting children with neurosensory disabilities across 12 age groups ranging from 12 months to 60 months [38]. One possible explanation for the variation in results could be due to lack of access to more rigorous clinical assessment tools in developing countries. In a study of the general population, Veldhuizen et al. found poor agreement between the ASQ and the Bayley Scales of Infant Development (BSID-III) with a sensitivity of 41% and specificity of 82%. Given Veldhuizen et al. used a 2SD cutoff for the ASQ, their results would only be comparable to the 2SD findings in this study [39].

Our fixed effects regression analysis revealed that communication, personal social and problem-solving scores correlated positively, and fine motor correlated negatively with age of the children. This finding is consistent with the relationship between established US cut-off values and age [25]. Moreover, the differences in domains scores between children with and without NDD increase with age, except for personal social scores (Fig. 2). This may reflect developmental delays becoming more noticeable when children are older, or the ASQ performing better when temporally closer to the age of diagnosis.

These findings offer important implications for practice and research. Waiting until school age for a child to demonstrate a lack of major milestones may not be as effective as an intervention within the first 3 years of life [40]. Using the ASQ as a valid and reliable [25, 28, 41] screening tool to exclude children who do not develop a NDD can help streamline early identification of children who should go through more intensive diagnosis assessment for NDD. An RCT suggests that early screening improves referral rates and time [42]. Children who were screened with the ASQ under 30 months of age were more likely to be identified with developmental delays, referred to early intervention, and eligible for early intervention services in a timelier fashion.

While the evidence on screening for developmental delay under the age of four is inconclusive [43], our findings from population survey data suggests that the 24 months ASQ with 1SD might be a valuable screening tool for identifying children at an early age who are at the high risk of developing a NDD. Inaccurately identifying a child as having a risk of NDD at a very young age could have adverse effects later in life and increase anxiety in parents [44, 45]. While our study showed that in general, the ASQ could be simple and valid screening tool to exclude children who do not develop NDD, we have to accept the risk of false-negatives. Tools with higher specificity come with greater risk of false negatives, which could have adverse consequences [45–47]. For those children who are identified as at risk of developing a NDD, appropriate developmental and comprehensive medical evaluations to determine a particular NDD are needed to prompt initiation of specific and appropriate early childhood therapeutic interventions.

### Strengths and limitations

The strength of our study is that we have used a large population-based survey, which improves the generalizability of our findings for the Canadian population. The ability to look at the longitudinal relationship of the ASQ domains scores over early development using fixed effect models which control effects of other domains provides insight into the distribution of domain scores at different time points. The study has three limitations. First, the ASQ-2 has been revised to the ASQ-3, which is now typically used in a clinical setting. There are minor differences in wording of items in the two versions and it is possible that this may have some effect on the study results when considering the ASQ-3, however the ASQ authors found few significant differences between the ASQ2 and ASQ3. A study by Kyerematen et al. [48] combined samples from the ASQ 2 and 3 suggesting comparable samples, however the version should be considered when interpreting these results. Second, only four domains of the ASQ were available in the dataset. To address this, we compared the sensitivity and specificity using four domains and all five domains in the cycle 4 data, which has ASQ scores on five domains and did not find any significant differences. Third, the classification of child health was limited to health conditions and behaviours reported by parents and not a clinical scale of behaviour problems.

## Conclusion

The high negative predictive value and specificity of the ASQ supports its potential use in identifying children who will not develop a NDD. While low sensitivity was found at most ages of development, the ASQ at 24 months with 1SD may be a good tool as a first step for early identification of children with NDD for early intervention. Further testing of its reliability and validity in a diverse population with all five domains and a clinical gold standard is needed to confirm our results.

## Additional file

Additional file 1: The flow chart for the selection of samples for the short and the long short. First, children with NDD were identified and followed them in the previous cycle(s) for ASQ scores. (DOCX 2 kb)

## Abbreviations

ASQ: Ages and Stages Questionnaire
HUI: Health Utilities Index
NDD(s): Neurodevelopmental Disorder(s)
NPV: Negative predictive value
PMK: A person most knowledgeable about the child
PPV: Positive predictive value
