# Supplementary material for: Using the ages and stages questionnaire in the general population as a measure for identifying children not at risk of a neurodevelopmental disorder
Source: BMC Pediatr. 2018 Apr 3;18:122. doi: 10.1186/s12887-018-1105-z (PMC5883588; doi:10.1186/s12887-018-1105-z)
Supplement: Supplementary file 1 — The flow chart for the selection of samples for the short and the long short. First, children with NDD were identified and followed them in the previous cycle(s) for ASQ scores. (DOCX 2 kb) [file 12887_2018_1105_MOESM1_ESM.docx]

**Additional file 1**

**Short Cohort**

Identify

Sample ASQ-2 Scores

4 to 5 years 2 to 3 years

**Long Cohort**

Identify

Sample ASQ-2 Scores ASQ-2 Scores

4 to 5 years 2 to 3 years 0 to 1 years
